# Hematopoietic cell transplantation and cell therapy activity landscape survey in the Kingdom of Saudi Arabia; a report from the Saudi Society of Blood and Marrow Transplantation (SSBMT)

**DOI:** 10.1038/s41409-024-02240-3

**Published:** 2024-03-08

**Authors:** On behalf of Saudi Society of Blood & Marrow Transplantation (SSBMT), Naila Shaheen, Ibrahim Abosoudah, Mohammad Alshahrani, Mohsen Alzahrani, Mohammed Essa, Bader Alahmari, Enas Mutaher, Solaf Kanfar, Ahmad Alsaeed, Sameer Alamoudi, Dania Monagel, Mohammed Marei, Musa Alzahrani, Abdulrahman Alsultan, Abdullah Aljefri, Ahlam Masari, Omer Alsharif, Ammar H. Alsughayir, Ayman Hejazi, Saad Aldaama, Ahmed Alaskar

**Affiliations:** 1 King Abdullah International Medical Research Center, Riyadh, Kingdom of Saudi Arabia; 2 King Saud bin Abdulaziz University for Health Sciences, Riyadh, Kingdom of Saudi Arabia; 3 King Faisal Specialist Hospital & Research Centre, Jeddah, Kingdom of Saudi Arabia; 4 Prince Sultan Military Medical City, Riyadh, Kingdom of Saudi Arabia; 5 Division of Adult Hematology and SCT, King Abdul-Aziz Medical City, Riyadh, Kingdom of Saudi Arabia; 6 Department of Pediatric Hematology Oncology, King Abdullah Specialist Children Hospital, Riyadh, Kingdom of Saudi Arabia; 7 Department of Hemato-oncology, King Fahad Specialist Hospital, Dammam, Kingdom of Saudi Arabia; 8 Princess Norah Oncology Center, King Abdulaziz Medical City, Jeddah, Kingdom of Saudi Arabia; 9 King Saud bin Abdul-Aziz University for Health Sciences, Jeddah, Kingdom of Saudi Arabia; 10 King Abdullah International Medical Research Center, Jeddah, Kingdom of Saudi Arabia; 11 King Fahad Medical City, Riyadh, Kingdom of Saudi Arabia; 12 King Saud University, Riyadh, Kingdom of Saudi Arabia; 13 King Faisal Specialist Hospital, Riyadh, Kingdom of Saudi Arabia

**Keywords:** Epidemiology, Haematopoietic stem cells

## Abstract

Hematopoietic Cell Transplantation (HCT) activity was surveyed in the Kingdom of Saudi Arabia (KSA). The overall rate of HCT per 10,000,000 inhabitants doubled every 10 years. 15,031 HCTs were reported by all the functional HCT centers in KSA since inception of HCT program. Out of total HCT 15,031; 10,232(68%) were reported in adults, and 4799(32%) in the pediatric population. Allogeneic HCT constituted 10,489(70%) of total HCT, with majority from Human Leukocyte Antigen matched identical sibling (85.4%). The autologous HCTs were 4542(30%). During the last five years 2018–2022; in total 5164 HCTs were performed, with the majority had allogeneic HCT 3,085(59.74%), followed by the autologous HCT 3085(40.2%). The top three main indications of the autologous HCT were Multiple Myeloma 299(28%), Hodgkin Lymphoma 293(27.8%), and Non-Hodgkin Lymphoma 212(20%). Hemoglobinopathies 615(27.6%) were mostly indicated for allogeneic HCT, followed by Acute Myeloid Leukemia 433(19.4%), and Precursors Lymphoid Neoplasms 322(14.4%). The HCT activity landscape survey provides the updated current state and trends for HCT in KSA. The reported HCT numbers differ than what was reported by international registries, since not all the cases have been reported. We urge to have a common data hub nationally in order to capture the actual number of cases.

## Introduction

The population of Kingdom of Saudi Arabia as of 2022 census data was 32.2 million [1]. As per the Saudi Cancer Registry Report, the incidence of Non-Hodgkin Lymphoma (NHL) was 5.9/100,000, Leukemia 5.1/100,000, Hodgkin Lymphoma (HL) 3.5/100,000, and Multiple Myeloma (MM) 2.1/100,000. Collectively the hematology cancer ranks first in males, while second in females [2]. A rise in lymphoma cases have been reported in both genders worldwide [3]. In the Kingdom of Saudi Arabia (KSA) HL has been reported to increase by gender [4]. Among non-malignant diseases; Sickle Cell Disease (SCD) prevalence in KSA was 0.26% in 2007 [5], which was reported as 145/10,000 cases in 2017, as well the prevalence in the Eastern province was reported to be higher [6].

Hematopoietic Cell Transplantation (HCT) is a curative therapy for several congenital, malignant/ non-malignant disorders, and acquired disease of the immune system [7]. Over the years the new advancement in the conditioning regimen and techniques have led to the better outcomes [8].

In KSA, the first HCT was done in 1984. The total number of reported HCTs were 6184; autologous HCT 4505(73%) and allogeneic HCT 1679(27%) in 2016 [9]. Since then HCT program and number of HCT has expanded in KSA. In this paper, we report the results of a survey pertaining to HCT activity in the Kingdom of Saudi Arabia over the last five years 2018–2022.

## Material and methods

The key expert leaders in the Hematology were invited to participate in the survey. The participating centers were identified by the key expert leaders. HCT activity landscape electronic survey was sent via an email to the adult/pediatric hematology consultants across KSA: Central, Western, and Eastern regions. Key expert leaders’ opinion was captured, along with the provided HCT related data. The data was extracted based on their provided information, along with the published literature.

The HCT activity landscape survey was composed of 25 questions related to: HCT set-up (*n* = 6), facility (*n* = 4), reporting to registries, clinical database (*n* = 3), availability of medications (*n* = 1), manpower (*n* = 6), type of procedures (*n* = 1), total number of HCT since inception (*n* = 1), number of HCT over five years (2018–2022) (*n* = 1), HCT indications (*n* = 1), number of received chimeric antigen receptor (CAR) T-cells therapies (*n* = 1), and the status of CAR T-cell therapy initiation (*n* = 1).

Data related to all centers in the KSA was aggregated and reported collectively. The supplementary data was utilized from the European Society for Blood and Marrow Transplantation (EBMT) data as applicable; HCT indications, and number of HCT over last five years. Indications for only first HCTs were considered. The number of HCT across years were compared with the EBMT data. The transplant rate was defined as the number of first transplants per 10 million inhabitants per year. The re-transplant information was not included. Data was analyzed using excel.

### Participating teams

The invited key expert leaders representing the HCT teams in three regions had participated in the survey. The HCT teams were located in the three regions (Central, Western, and Eastern). Each team included both adult and pediatric units.

## Results

### HCT activity landscape survey in KSA 2022

In KSA, the HCT activity was started in 1984, having in total eight centers across KSA until date. Three centers have become functional in year 1984, 1996, and 2001 respectively. Five centers have become functional during the last decade, since 2010 to 2018. The distribution of HCT centers and beds per regions: Central region (5 centers; bed capacity 77), Western region (2 centers; bed capacity=41), and Eastern region (1 center; bed capacity=18). Three centers had reported accreditation either by the Foundation for the Accreditation of Cellular Therapy (FACT) or Joint Accreditation Committee ISCT-Europe & EBMT (JACIE). Other centers are in a process of acquiring accreditation.

**Fig. 1:**
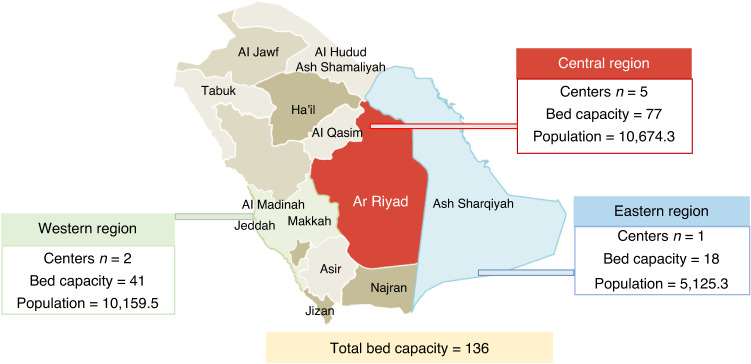
Distribution of HCT centers across KSA. The figure displays the distribution of HCT centers across three regions in KSA along with the bed to population ratio.

The population to bed ratio was, central region (7.21 per one million), Western region (4.03 per one million), and Eastern region (3.51 per one million) (Fig. 1). The key expert leaders had reported data contribution mainly to international registries; EBMT (75%), followed by Center for International Blood and Marrow Transplant Research (CIBMTR) (37%), and Eastern Mediterranean Blood and Marrow Transplantation (EMBMT) (12%), and a local registry; Saudi Bone and Marrow Transplant Registry (SBMTR) (12%).

**Fig. 2:**
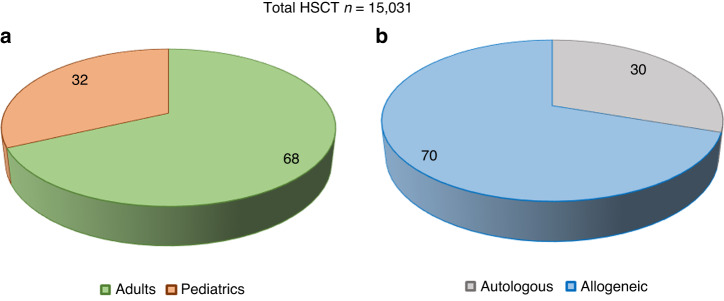
Distribution of HCT by age groups and HCT types. **a** Displays proportion of HCT by Adult and Pediatric age groups; **b** displays distribution of HCT by types (Allogeneic vs. Autologous).

A total of 15,031 first HCT procedures were performed since inception of HCT program in 1984. Allogeneic HCT constituted majority 10,489(70%), the autologous were 4542(30%). Out of total 15,031; 10,232 (68%) HCTs were reported in adults, and 4799(32%) in the pediatrics age group (Fig. 2a, b).

**Fig. 3:**
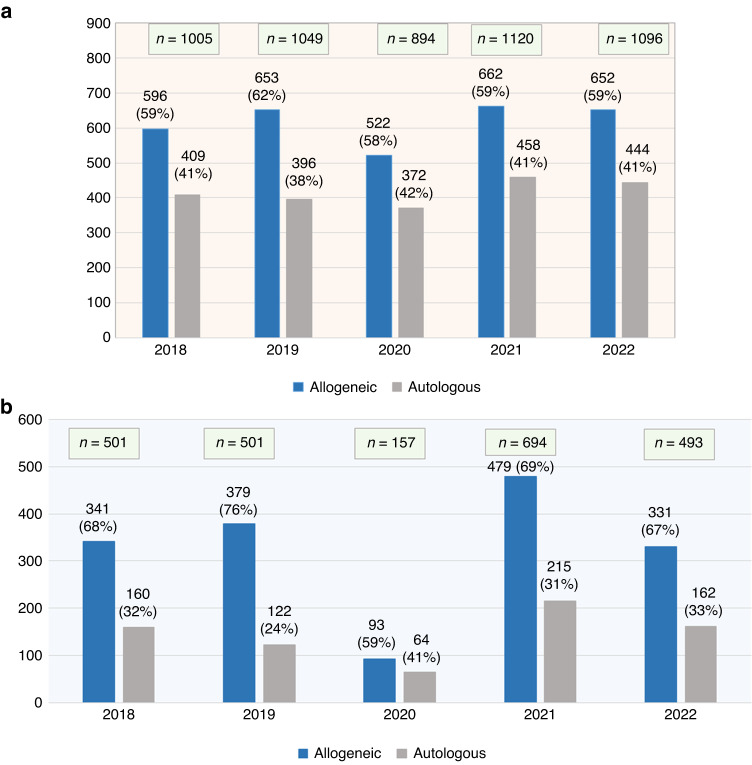
Distribution of HCT by types over 5 years. **a** Displays distribution of HCT by type (Allogeneic vs. Autologous) over 5 years 2018–2022 based on the landscape survey data; **b** displays distribution of HCT by type (Allogeneic vs. Autologous) over 5 years 2018–2022 based on the EBMT data. The EBMT supplemental data used in this paper were based on a single overview per year, without incorporating important qualified updates made retrospectively, further to the impact of the COVID-19 pandemic on reporting. This may have resulted in significant under-reporting of HSCTs in the EBMT data.

Figure 3a, displays the number of first HCT by type over five years 2018–2022. In total, 5164 HCTs were performed during last five years. There is a swift increase in the number of HCTs over the years. Figure 3b, displays the number of the HCT over the years as per the data acquired by EBMT.

**Fig. 4:**
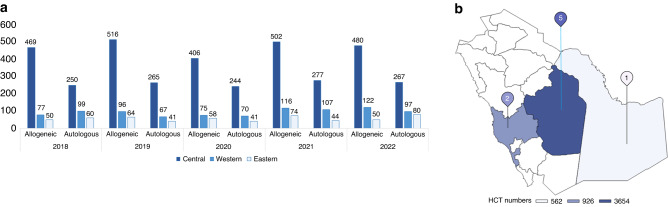
Distribution of HCT by regions in KSA. **a** Displays distribution of HCT numbers by regions and types (Allogeneic vs. Autologous) over 5 years (2018–2022); **b** displays distribution of total HCT numbers by regions over 5 years (2018–2022). (*Central region; 5 centers, Western region; 2 centers, Eastern region; 1 center).

**Fig. 5:**
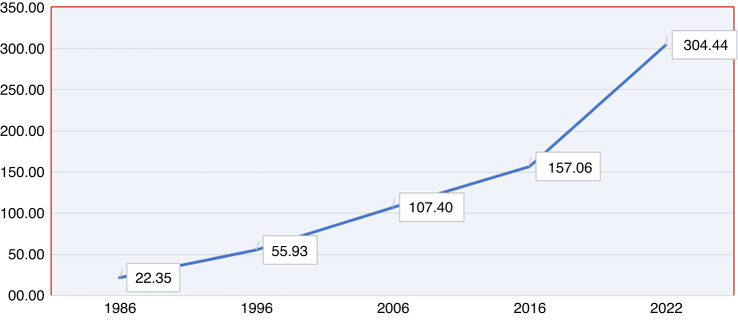
Rate of HCT activity in KSA. The figure displays overall rate of HCT since initiation of transplant activity in KSA over the years (1986–2022).

The major bulk of HCT activity pertains to the Central region (Fig. 4). The rate of HCT since the initiation of transplant activity in KSA is shown in Fig. 5. The overall rate of transplantation performed per 10,000,000 for the year of 2016 was 157.06, which is 7 times that of 1986. The overall trend was doubling every 10 years since 1986 up to 2006 after which it slowed down to only (46%) in 2016 compared to that of 2006. However, there is an exponential rise in HCT between 2016 and 2022 (Fig. 5).

### Allogeneic hematopoietic cell transplantation

**Fig. 6:**
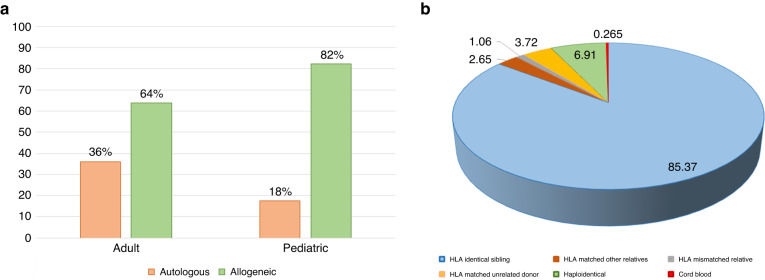
Distribution of HCT and donor types. **a** Displays distribution of HCT types (Allogeneic vs. Autologous) by age groups (Adult vs. Pediatric); **b** displays distribution of types of donor. (*Data source is Saudi Blood and Marrow Transplantation Registry (SBMTR)).

The allogeneic HCT was higher in pediatric group (Fig. 6a). Full Human Leukocyte Antigens (HLA) match was the most common donor type (85%) for the allogeneic HCT (Fig. 6b). As depicted in the Fig. 4a, overall the allogeneic HCT is higher than autologous throughout the years 2018–2022. The donor sources were HLA identical siblings (85.4%), Haploidentical (6.9%), HLA matched unrelated donor (MUD) (3.7%), HLA matched related (2.7%), HLA mismatched related (1.1%), and cord blood (0.3%).

**Fig. 7:**
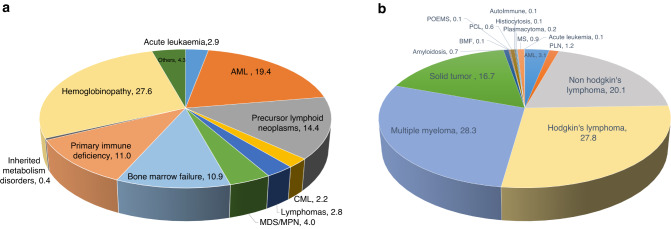
Indications by types of HCT. **a** Displays indications of Allogeneic HCT (*Data source EBMT); **b** displays indications of Autologous HCT. (*Data source EBMT).

**Fig. 8:**
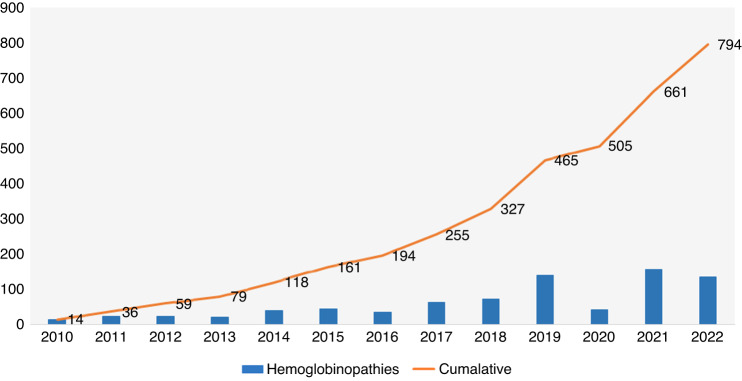
HCT indication over the years. The figure displays change in HCT indication; hemoglobinopathies over the years (2010–2022). (*Data source EBMT).

Fig. 7(a) summarizes indications of allogeneic HCT (*n* = 2230). Top three indications were Hemoglobinopathies 615 (27.6%), Acute Myeloid Leukemia (AML) 433(19.4%), and Precursors Lymphoid Neoplasms 322(14.4%). Hemoglobinopathies have a swift increase over the years, as an indication of allogeneic HCT (Fig. 8).

### Autologous hematopoietic cell transplantation

Top three indications of autologous HCT (*n* = 1055) were Multiple Myeloma (MM) 299(28.3%), HL 293(27.8%), and NHL 212(20%) (Fig. 7b).

### Stem cell donor registries & cord blood banks (CBBs)

Based on the HCT landscape survey, two stem cell donor registries were identified; both are members of WMDA, and have in total of more than 160,000 registered donors [10].

Both registries have the affiliated two Cord Blood Banks (CBBs); both are FACT accredited, and member of WMDA. In total, both CBBs have 10,000 cryopreserved units, and as well have facilitated more than 130 stem cell donations [11].

### CAR T-cell therapy 

The use of CAR T-cell therapy is summarized in Table 1. The CAR T-cell therapy has become functional in three centers. So far in total 70 recipients have received the CAR T-cell therapy of which 46 were adults and 24 pediatric.

**Table 1:** CAR T-cell therapy in KSA.

CAR T-cell therapy	Initiated in 3 centers
Indications	Acute Lymphoblastic Leukemia, Lymphoma, Multiple Myeloma
Received CAR T-cell therapy types	Ciltacabtagene Autoleucel (Cilta-cel), Tisagenlecleucel (Tisa-cel), Axicabtagene Ciloleucel (Axi-cel)
Number of CAR T-cell therapies	Adult *n* = 46
	Pediatrics *n* = 24

## Discussion

This is a first survey reporting HCT landscape in KSA collectively over the last five years (2018–2022). The data demonstrates the transplant activity in KSA at the national level. It describes the progress in HCT since the initiation of the transplant program, and the way forward.

The results show accelerated growth in the number of HCT transplantation per 10,000,000 inhabitants over the past 30 years since HCT program inception, with the highest level in the Central region. The majority of the centers exist in the Central region, explaining the highest number of HCT reported in the Central region. However, more centers are needed based on the bed to population ratio per each region, which aligns with the distribution of beds in general population, as reported by a study published in KSA 2022. The total number of beds over the years 2015–2019 increased in the general population from 3469 per 1,576,070 population to 3849 per 1,710,908 population. In total the rate of beds increased from 237 (2015) to 246.8 (2019) [12]. The results of the HCT activity landscape survey showed that rate of HCT has increased over the years, which is in accordance with the global increase in the HCT numbers; (USA) [13], Europe [14], and China [15]. Several reasons attribute to this global increase in HCT (i) advancement in the HCT methods, and techniques (ii) advancement in the conditioning regimen (iii) shift in disease indication; considering non-malignant indications, and early stage malignant diseases [15], and (iv) inclusion of more donor types. The increasing number of centers performing HCT since 1984, from a single center to 8 centers as of 2022 is also attributed to the swift increase in the healthcare expenditure since 2011 in KSA [16].

As per the HCT activity landscape survey in KSA, both autologous and allogeneic HCT has increased overtime, however the rate of allogeneic is higher than the autologous. The rate of allogeneic is also reported as higher than autologous in China, [15] and in the Eastern Mediterranean region [17]. On the contrary, as per the transplant activity in USA 2018, autologous HCT were higher in adults (65%) [18], as well higher autologous HCT were reported by EBMT [14]. The potential rationale of higher allogeneic HCT compared to autologous for both adults and pediatric age groups in KSA is the chance of finding a matched sibling donor. Consanguinity is helpful in getting matched related identical donors. The reported reason of a high likelihood (68%) of finding a matched sibling donor in Saudi adults is due to the bigger family size [19].

The number of HCT over five years in our survey differ than what was reported by EBMT. One possible reason of less HCT numbers in EBMT data is that only (75%) of the respondents had reported contribution to the EBMT. One local registry SBMTR exists but only (12%) of the participants had reported data submission to the SBMTR. The HCT percentage decreased by (15%) in year 2020 (COVID outbreak), and increased by (25%) in 2021.

The most common indications of autologous HCT in Saudi population was MM, HL, and NHL similar to what was reported in China: MM, NHL, Acute Myeloid Leukemia (AML) [15], EBMT: (Plasma Cell Disorders (PCD), NHL, HL) [14], and Latin America: PCD, NHL, and HL [20].

The most common indication of allogeneic HCT was hemoglobinopathies, AML, and precursor lymphoid neoplasms in Saudi population, similar to what was reported in China: AML, Acute Lymphoblastic Leukemia (ALL), Aplastic Anemia (AA) [15], EBMT: AML, ALL, MDS [14], and Latin America: AML, ALL, Bone Marrow Failure (BMF) [20].

The indications for allogeneic HCT as reported in KSA 2016 were AML (20%), ALL (18%), and Chronic Myeloid Leukemia (CML) (16%) [9]. While the current survey shows there has been a switch in indication of allogeneic HCT from CML (16%) [9] to hemoglobinopathies (27.6%) in KSA over the last five years. A possible explanation is a high prevalence of hemoglobinopathies (sickle cell disease 0.26%) in the Eastern region [21]. KSA has a young median age of transplant patients [9], which aligns with the Saudi population’s relatively young average age (29 years) [22].

The CAR T-cell therapy was initiated in 2019. Currently CAR T-cell therapy is reported to be functional in three centers, for indications ALL, and lymphomas. The other centers have initiated the process and will be in the list soon. This will likely lead to a reduction in the rate of HCT and increase in the CAR T-cell therapies in upcoming years. Three products are currently being used in three centers i.e. Cilta-cel, Tisa-cel, and Axi-cel. The CART-cell in-house production is ongoing as well.

### Registries in the Kingdom of Saudi Arabia

There are two donor registries; Saudi Stem Cell Donor Registry (SSCDR) and King Faisal Specialist Hospital & Research Center Donor Registry (KFSH&RC DR) along with two cord blood banks; KAIMRC CBB and KFSH&RC CBB. Currently, the HCT centers submit the data to the international registries. However, for HCT data capturing locally one registry, Saudi Bone and Marrow Transplant Registry (SBMTR) has been established since 2015.

### Limitations

The HCT cases are a case mix and do not represent exactly region of origin; a subject from one region having transplant in another region, or residing in another region. We did not capture the outcome and the donor data. Lastly, data heterogeneity due to no one source of data capturing still prevails as a challenge.

## Conclusion

The HCT activity landscape survey provides the updated current state and trends for HCT in KSA. In summary, the HCT numbers have increased in the Kingdom of Saudi Arabia. The HCT standards, quality of care, and practices match with the internationally renowned HCT centers.

Based on the HCT activity landscape survey, we recommend (i) an urge to have a national data hub in order to capture the HCT and cellular therapy activities, (ii) to increase the homogeneity of the reported data, (iii) to promote collaboration at national and international levels, (iv) we also need to expand current capacity to accommodate the HCT & cellular therapy needs at the national level, and (v) to establish local CART-Cell production (academic production vs. local manufacturing), and (vi) lastly to have a periodic survey in order to capture the updated numbers and HCT outcomes.

## Data Availability

The datasets generated and/or analyzed during the current study are not publicly available due to the institutional rules and regulations but are available from the author on a reasonable request.
