# Correction: Hematopoietic cell transplantation and cell therapy activity landscape survey in the Kingdom of Saudi Arabia; a report from the Saudi Society of Blood and Marrow Transplantation (SSBMT)

**DOI:** 10.1038/s41409-024-02450-9

**Published:** 2024-11-04

**Authors:** On behalf of Saudi Society of Blood & Marrow Transplantation (SSBMT), Naila Shaheen, Ibrahim Abosoudah, Mohammad Alshahrani, Mohsen Alzahrani, Mohammed Essa, Bader Alahmari, Enas Mutaher, Solaf Kanfar, Ahmad Alsaeed, Sameer Alamoudi, Dania Monagel, Mohammed Marei, Musa Alzahrani, Abdulrahman Alsultan, Abdullah Aljefri, Ahlam Masari, Omer Alsharif, Ammar H. Alsughayir, Ayman Hejazi, Saad Aldaama, Ahmed Alaskar

**Affiliations:** 1 King Abdullah International Medical Research Center, Riyadh, Kingdom of Saudi Arabia; 2 King Saud bin Abdulaziz University for Health Sciences, Riyadh, Kingdom of Saudi Arabia; 3 King Faisal Specialist Hospital & Research Centre, Jeddah, Kingdom of Saudi Arabia; 4 Prince Sultan Military Medical City, Riyadh, Kingdom of Saudi Arabia; 5 Division of Adult Hematology and SCT, King Abdul-Aziz Medical City, Riyadh, Kingdom of Saudi Arabia; 6 Department of Pediatric Hematology Oncology, King Abdullah Specialist Children Hospital, Riyadh, Kingdom of Saudi Arabia; 7 Department of Hemato-oncology, King Fahad Specialist Hospital, Dammam, Kingdom of Saudi Arabia; 8 Princess Norah Oncology Center, King Abdulaziz Medical City, Jeddah, Kingdom of Saudi Arabia; 9 King Saud bin Abdul-Aziz University for Health Sciences, Jeddah, Kingdom of Saudi Arabia; 10 King Abdullah International Medical Research Center, Jeddah, Kingdom of Saudi Arabia; 11 King Fahad Medical City, Riyadh, Kingdom of Saudi Arabia; 12 King Saud University, Riyadh, Kingdom of Saudi Arabia; 13 King Faisal Specialist Hospital, Riyadh, Kingdom of Saudi Arabia

**Keywords:** Epidemiology, Haematopoietic stem cells

Correction to: *Bone Marrow Transplantation* 10.1038/s41409-024-02240-3, published online 08 March 2024

In this article a Clarification Statement has been added to the caption of Fig. 3.

It should read: The EBMT supplemental data used in this paper were based on a single overview per year, without incorporating important qualified updates made retrospectively, further to the impact of the COVID-19 pandemic on reporting. This may have resulted in significant under-reporting of HSCTs in the EBMT data.

